# The effect of maternal education on infant mortality in Ethiopia: A systematic review and meta-analysis

**DOI:** 10.1371/journal.pone.0220076

**Published:** 2019-07-29

**Authors:** Girmay Tsegay Kiross, Catherine Chojenta, Daniel Barker, Tenaw Yimer Tiruye, Deborah Loxton

**Affiliations:** 1 Department of Public Health, College of Health Sciences, Debre Markos University, Debre Markos, Ethiopia; 2 Research Centre for Generational Health and Ageing, Faculty of Health and Medicine, University of Newcastle, Newcastle, New South Wales, Australia; 3 School of Medicine and Public Health, Faculty of Health and Medicine, University of Newcastle, Newcastle, New South Wales, Australia; CUNY School of Public Health, UNITED STATES

## Abstract

**Introduction:**

Some studies in developing countries have shown that infant mortality is highly associated with maternal education, implying that maternal education might play an important role in the reduction of infant mortality. However, other research has shown that lower levels of maternal education does not have any significant contribution to infant survival. In this systematic review, we focus on the effect of different levels of maternal education on infant mortality in Ethiopia.

**Methods:**

MEDLINE, EMBASE, CINAHL, Scopus, and Maternity and Infant Care databases were searched between November 15, 2017 and February 20, 2018. All articles published until February 20, 2018 were included in the study. The data extraction was conducted in accordance with the Preferred Reporting Items for Systematic Review and Meta-Analyses (PRISMA 2009) guidelines. An I^2^ test was used to assess heterogeneity and a funnel plot was used to check publication bias.

**Findings:**

We retrieved 441 records after removing duplications. During screening, 31 articles were fully accessed for data extraction. Finally, five articles were included for analysis. The overall pooled estimate indicated that attending primary education was associated with a 28% reduction in the odds of infant mortality compared to those infants born to mothers who were illiterate, OR: 0.72 (95% CI = 0.66, 0.78). Another pooled estimate indicated that attending secondary education and above was associated with a 45% reduction in the odds of infant mortality compared to those infants born to mothers who were illiterate, OR: 0.55 (95% CI = 0.47, 0.64).

**Conclusion:**

From this study, understanding the long-term impact of maternal education may contribute to reduce infant mortality. Therefore, policy makers should give more attention in promoting the role of women through removing institutional and cultural barriers, which hinder women from access to education in order to reduce infant mortality in Ethiopia.

## Introduction

The infant mortality rate is one of the most important indices for gauging the overall level of public health and the social and economic development of a country or region [1]. At the beginning of the Sustainable Development Goals (SDGs) era in 2016, maternal and child mortality remained unacceptably high, especially in sub-Saharan Africa countries [2]. In the last two decades there has been a global underinvestment in the health of newborns and infants, and as a result of this, millions of preventable deaths occur each year [3]. At the end of the Millennium Development Goals in 2015, despite the policy support provided during this period, 4.5 million infant deaths occurred globally; and most of these deaths were in developing countries [4].

Africa has the highest infant mortality rate of all the regions of the world. According to the World Health Organization report in 2015, the global infant mortality rate was 32 per 1000 live births compared to the Sub-Saharan Africa region rate of 56 per 1000 live births [1].

The infant mortality rate in Ethiopia is one of the highest in Africa [5, 6]. According to the 2016 Ethiopian Demographic and Health Survey report, infant mortality was 48 per 1000 live births [5, 6]. Nevertheless, the infant mortality rate in Ethiopia has shown a 50% reduction in the last 16 years [6]. Several studies in developing countries have shown that infant mortality is highly associated with maternal education [7–9]. According to social science researchers, maternal education is one of the most important determinants of infant mortality [10–12]. Education is proposed to increase a mother’s knowledge of health care practices related to contraceptive utilization, nutrition, hygiene and disease prevention [13]. Education is also an important determinant of social and economic development and is strongly associated with various socioeconomic measures such as lifestyle, income, and fertility for both individuals and society [6, 13].

In Ethiopia, 51% of females age six and over have ever attended school and primary school is the highest level of education completed or attended for the majority of women [6]. In the 2016 Ethiopian Demographic and Health Survey report, only 4% of women completed secondary school or went beyond secondary school [6].

Educated mothers have been found to use information in a more effective way when caring for children and tend to seek appropriate health care more effectively than mothers who are not educated [14]. Research conducted in Nigeria showed that education has also been found to enable mothers to be more autonomous, to resist harmful traditional influences and practices, and to make economic decisions to spend more on caring for children [15]. Another research conducted in in India and Pakistan indicated that eeducated mothers are also more likely to change traditional practices such as feeding and childcare practices, and make changes in fertility preferences [16]. Furthermore, maternal education increases women’s involvement in family decisions and increases demand for health services such as antenatal care utilization and institutional delivery [17]. Educated mothers are also likely to carry out key components of newborn care such as weighing the infant at birth and not giving a prelacteal feed [18]. Maternal education is also more likely to generate income for the family and increase total family resources [19]. This may be through employment, as educated women are more likely to have paid work than uneducated women [20].

Despite the reported benefits of maternal education, some research in India and Ethiopia has shown that primary school and informal education does not have any significant contribution to the survival of infants or children [21, 22]. These findings suggest that level of education is an important factor to consider for infant and child survival, particularly since most women in Ethiopia who are educated are not educated beyond primary school level [6]. In the following systematic review, we focus on the effect of maternal education on infant mortality in Ethiopia. This systematic review is limited to Ethiopia because the categorization of education levels vary from nation to nation, which may prevent coherent results. Reviewing the effect of maternal education in one nation where infant mortality is a serious problem, namely Ethiopia, might provide important information, which can help for policy development in the nation. Therefore, the aim of this systematic review is to assess the effect of different levels of maternal education on infant mortality in Ethiopia.

## Method

### Search strategy

Several electronic databases were searched including MEDLINE, EMBASE, CINAHL, Scopus, and Maternity and Infant Care. The article search was started on November 15, 2017 and ended on February 20, 2018. We used a combination of multiple key terms including determinant, risk factor, child, infant, perinatal, neonatal, mortality, educational status, maternal education, education, mother, maternal, and Ethiopia. A search strategy was developed in the MEDLINE database and applied to the other electronic databases (see S1 Table). We also manually searched the reference lists of included articles.

### Inclusion and exclusion criteria

Journals, describing observational studies conducted in Ethiopia (case control, cohort and analytical cross sectional studies) that assessed the relationship between maternal education and infant mortality were included. In this systematic review, infant mortality was defined as death of a child less than one year of age. All papers published until February 20, 2018 were included in this review. Death of child less than 28 days old (neonatal mortality) and death of child less than five years old (child mortality) was excluded from the study. Research conducted outside of Ethiopia was also excluded from the study.

### Assessment of methodological quality

Manuscripts selected for analysis were assessed by two independent reviewers (GK, TT). For methodological validity, standardized critical appraisal instruments from the Joanna Briggs Institute Meta-Analysis of Statistics Assessment and Review Instrument (JBI-MAStARI) were used [23]. The critical appraisal checklists used in this study were for observational studies, such as analytical cross sectional studies [24], cohort [25] and case control [26] studies (see Table 1). Any disagreements that arose between reviewers were resolved through discussion. We did not exclude papers based on the methodological quality due to very few papers being eligible for the final analysis.

### Data extraction

The data extraction was conducted in accordance with the Preferred Reporting Items for Systematic Review and Meta-Analyses (PRISMA 2009) guidelines [27]. The relevance of each study was checked based on its title, objectives, and methodology. Firstly, articles were excluded based on their title. Then, abstracts were assessed and excluded if they did not match the current study objectives. For the remainder, the entire content of the articles was accessed and selected based on the exposure (maternal education) and dependent outcome (infant mortality) under review. Data extraction included specific details about the study design (cross sectional, case control and cohort), the sample size, and the number of events among exposure (the number of infant deaths among women with primary education and the number of infant deaths among women with secondary education and above) (see Table 1).

**Table 1:** List of studies included to show the effect of maternal education on infant mortality in Ethiopia.

Authors	Study design and setting	Study population and sample size	Education category	Methodological quality
Asefa M et al, 2000 [8]	Community based cohort study	8273 infants’ mothers were interviewed	No Education (no formal education) Primary education (Grade 1 to Grade 8) Secondary and above (Grade 9 and above)	9/11
Muluye S. & Wencheko E.,2005 [29]	Community based cross-sectional study	9861 children’s mothers were interviewed	No Education Primary education Secondary and above	7/8
Dube L et al, 2013 [30]	Community based matched case control study	254 infants’ mothers were interviewed	Illiterate Primary Secondary and above	10/10
Alemayehu et al, 2015 [31]	Population based cross-sectional study	A total of 16,267 women with 25,472 births	No Education Primary education Secondary and above	8/8
Kumar PP. and File G. 2015 [32]	Population based cross-sectional study	14,070 children mother were interviewed	illiterate Primary Secondary & above	6//8

### Data analysis

Information extracted from each original study was analyzed using RevMan5 software (include citation information here as per journal guidelines). A Preferred Reporting Items for Systematic Reviews and Meta-Analysis (PRISMA) checklist was used to report the results of the study [28]. A Funnel plot asymmetry in the random effects model was used to check for publication bias. The summary effect measure used was an odds ratio. We used the I^2^ and chi squared test to examine the heterogeneity of studies. The assumption of heterogeneity is there is no difference among studies, and if the value of I^2^ is greater than 70%, it indicates that there is a substantial variability among the studies.

## Results

### Search result

**Fig 1:**
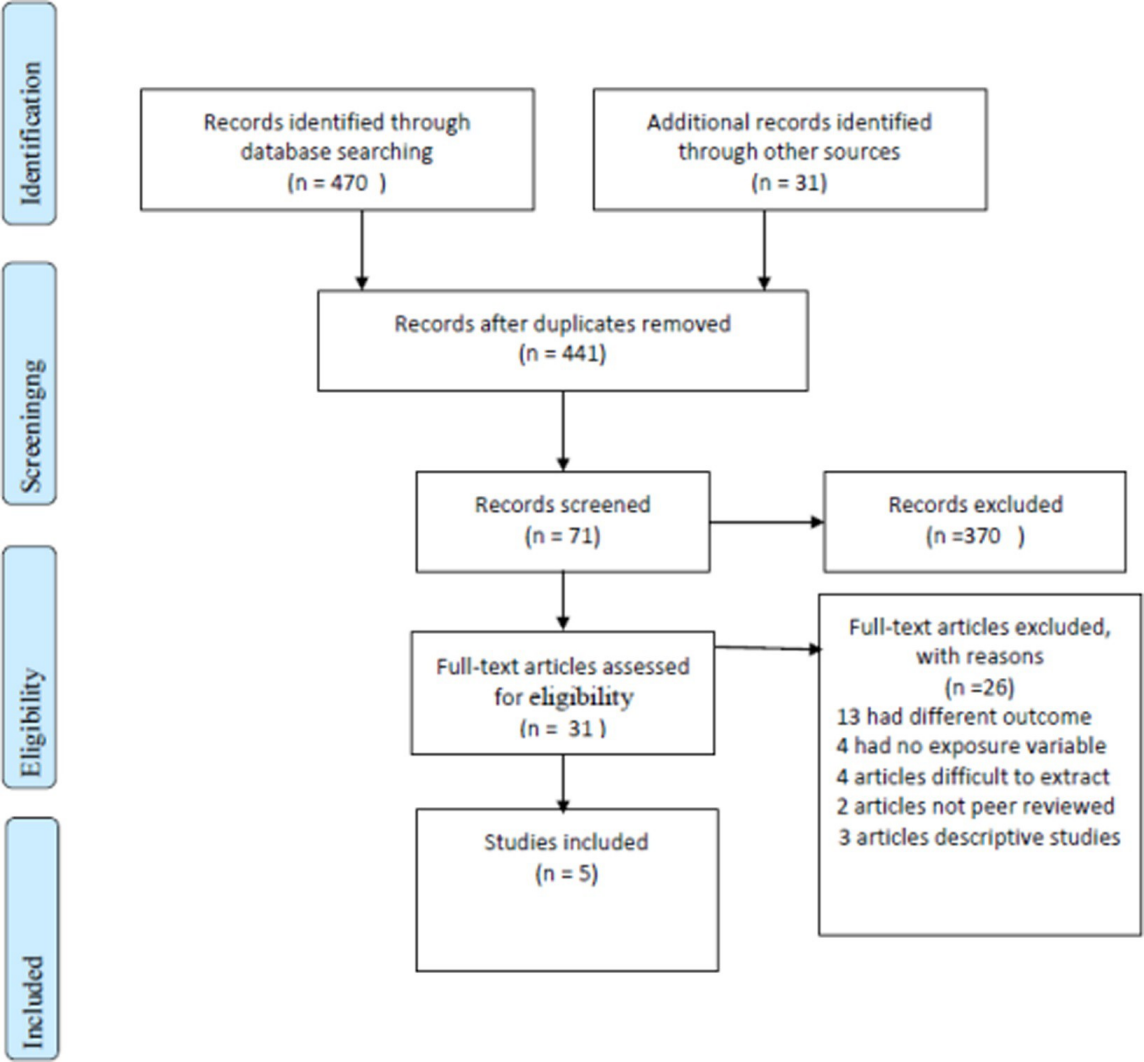
PRISMA flow diagram showing the procedure of selecting studies for meta-analysis in Ethiopia.

We retrieved 470 records from the database search and 31 records using hand searches from the reference lists of all previous articles. After removal of duplicate articles, 441 records remained. From these, 370 records were excluded in the initial screening because their title was not related to this systematic review. For the remaining 71 articles, abstracts were accessed and screened. From these, 40 articles were excluded due to the irrelevance of the exposure and outcome. From these remaining 31 articles, thirteen were excluded due to the outcome variable being different, such as under-five mortality and neonatal mortality. Four articles were excluded due to the exposure not being according to inclusion criteria. Another four articles were also excluded as the authors reported only the odds ratio, making it impossible to extract the data necessary for this analysis. We tried to communicate the authors through their email address to access the data, with no response. Five articles were also excluded due to not being peer reviewed and for reporting descriptive statistics only. Finally, five articles were included for analysis (Fig 1).

### Effect of primary education on infant mortality

**Fig 2:**
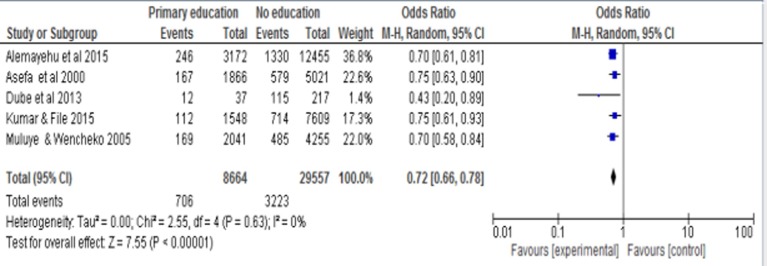
Forest plot of five studies to show the effect of primary maternal education on infant mortality in Ethiopia.

The overall pooled odds ratio estimate was 0.72 (95% CI = 0.66, 0.78). This pooled estimate indicated that attending primary education was associated with a 28% reduction in the odds of infant mortality compared to those infants born to mothers who were illiterate (Fig 2).

**Fig 3:**
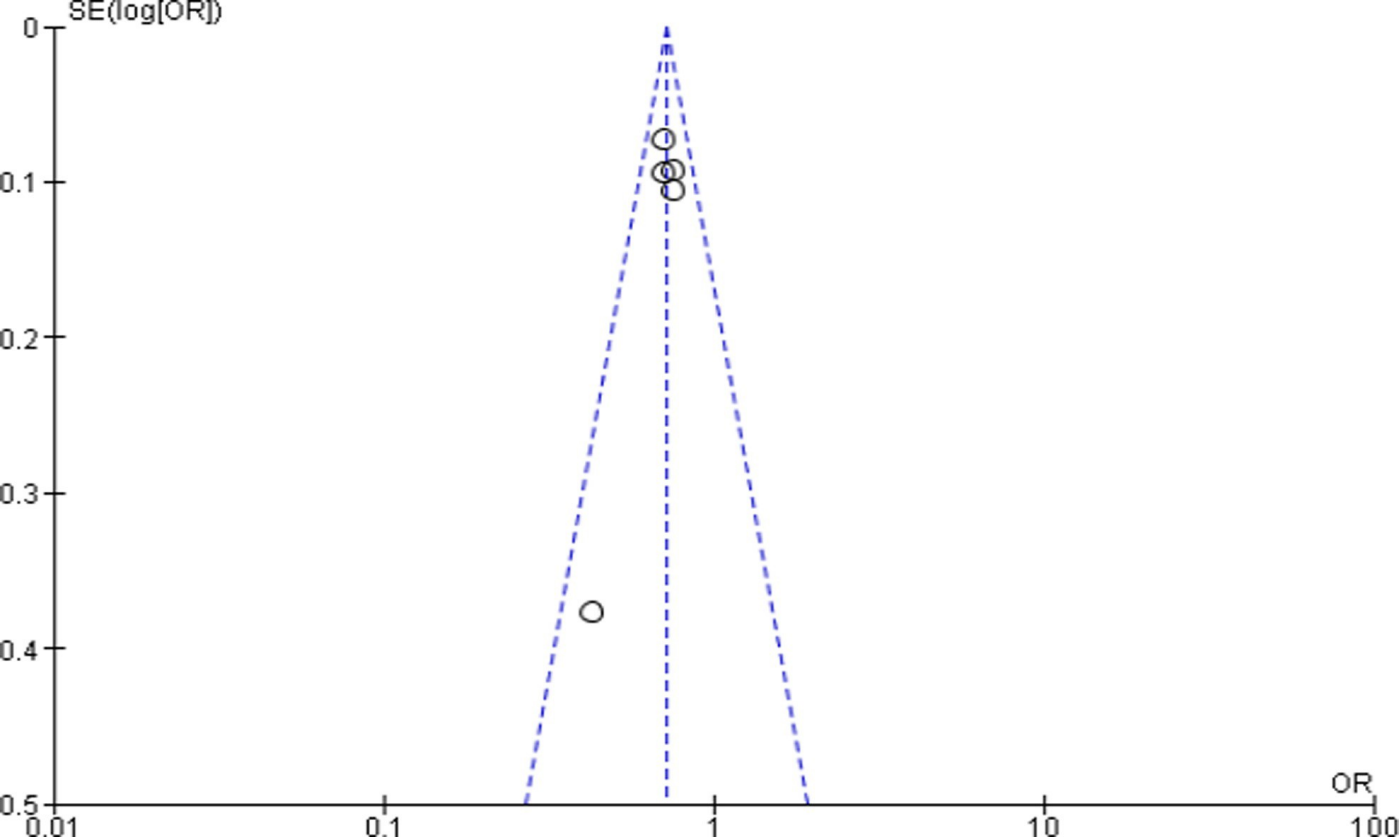
Funnel plot of five studies to show the effect of primary maternal education on infant mortality in Ethiopia.

The funnel plot showed that there is no evidence of publication bias (Fig 3). Regarding heterogeneity, there was no significant difference among the five studies (p = 0.63) indicating that there is no significant variability among the studies.

### Effect of secondary education and above on infant mortality

**Fig 4:**
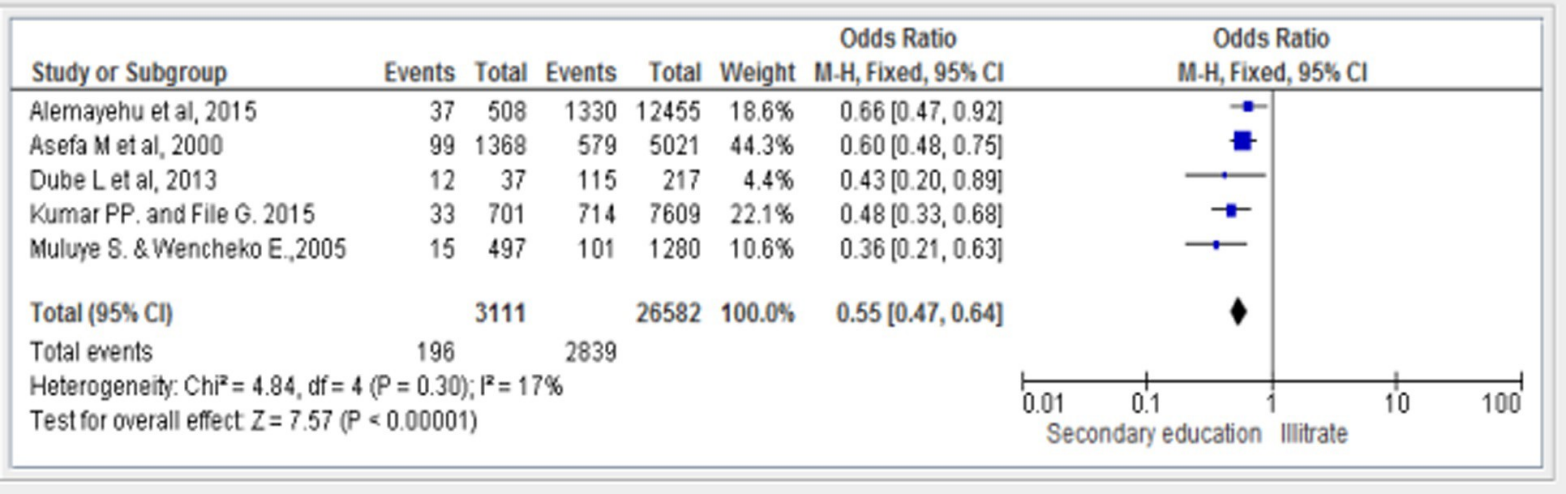
Forest plot of five studies to show the effect of secondary and above maternal education on infant mortality in Ethiopia.

The overall pooled odds ratio for the effect of secondary education was 0.55 (95% CI = 0.47, 0.64). This pooled estimate indicates that attending secondary education and above was associated with a reduction in the odds of infant mortality by 45% compared to those infants born to mothers who were illiterate (Fig 4).

**Fig 5:**
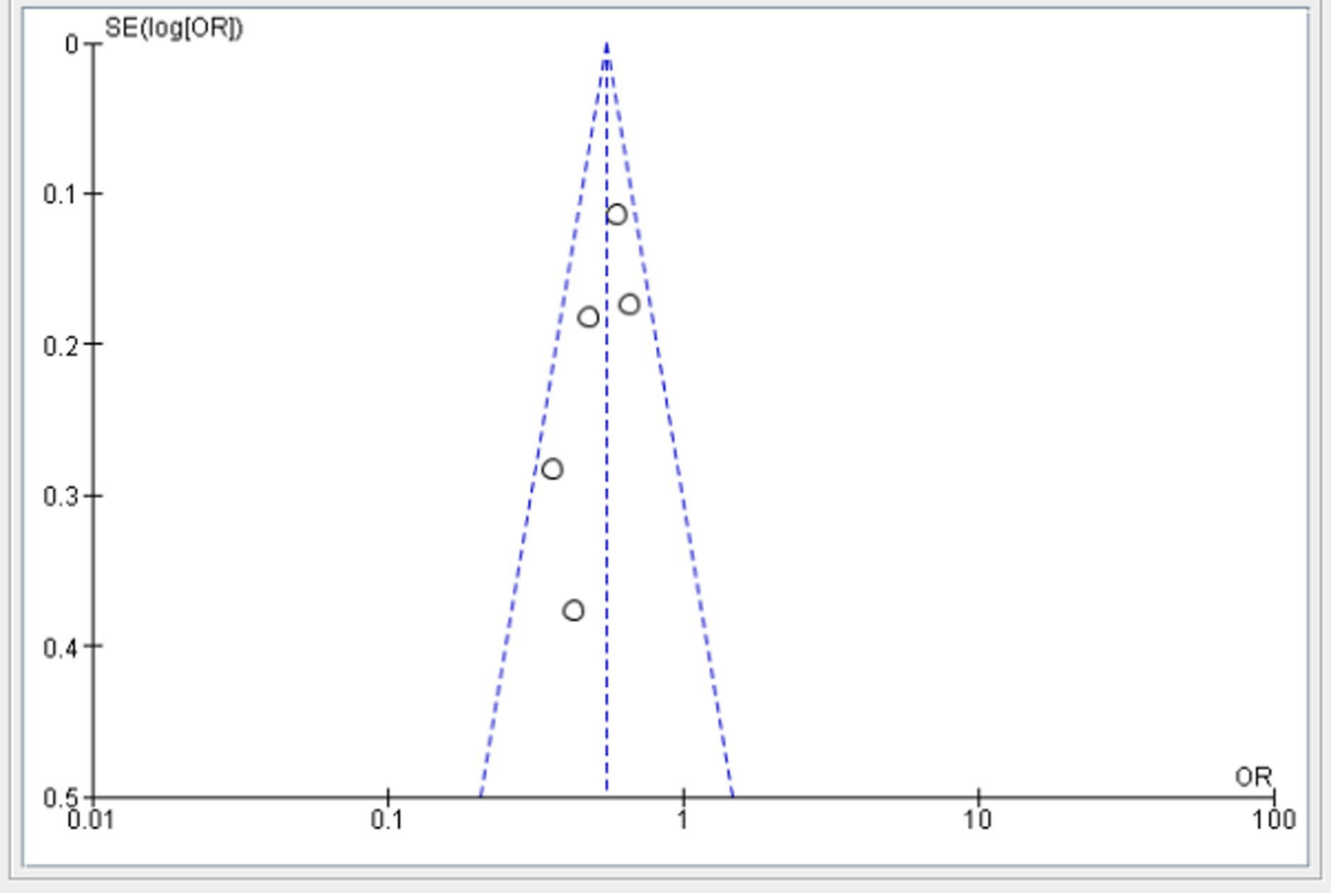
Funnel plot of five studies to show the effect of secondary and above maternal education on infant mortality in Ethiopia.

The funnel plot showed that there is no evidence of publication bias (Fig 5). Regarding heterogeneity, there is no significant difference among the five studies (P = 0.30) and I^2^ of 17% indicating that there was no significant variability among studies.

## Discussion

The overall pooled estimate from the meta analysis showed that there is a strong association between maternal education and infant mortality. The pooled odds ratio for primary education was 0.72 (95% CI = 0.66, 0.78) and the pooled odds ratio for the effect of secondary education on infant mortality was 0.55 (95% CI = 0.47, 0.64).

In Ethiopia, mortality has been found to be higher for infants born to mothers who were illiterate than those whose educational status was secondary and above [33]. The risk of infant death was 3.8 and 3 times higher among mothers who had no education and attended primary education respectively compared to illiterate mothers [29]. In a cross sectional survey from two rounds of nationally representative data among 45,952 live births, it was shown that a mother who completed high school was 31% less likely to experience the death of an infant than a mother with no education [31]. In a study in Addis Ababa from a population and housing census, researchers also reported that child survival is greater among educated mothers and the impact of maternal education was generally more important in the step from primary to secondary schooling than from the illiterate to the literate group [34]. In addition, researchers in Ethiopia found that children whose mothers have attended secondary or higher education were 42% less likely to die as compared with infants whose mothers have no education [35]. In a longitudinal population-based surveillance in Ethiopia researchers also found that infants had a 56% lower risk of death if they were born to mothers who attained secondary school and above compared to those infants whose mothers did not attend formal education [21]. In the 2016 Ethiopian Health Survey report, the rate of infant mortality declined by 29 deaths per 1,000 live births (from 64 deaths per 1000 livebirths to 35 deaths per 1000 live births as maternal education increased to secondary school) [6].

The association between education and infant health can be explained a number of different ways [36, 37]. An educated mother might be more able to use health care information and therefore understand the importance of child immunization, early child health seeking and appropriate caring for children practice better than illiterate mothers [36]. An educated mother can also seek appropriate health care, can be autonomous, could refuse harmful traditional practices and could make economic decisions to spend more on caring for children [15]. Educated mothers can modify the traditional child feeding and care practices, changing fertility preference such as giving more focus to the quality of children’s lives rather than quantity, and increase their involvement in family decisions [19, 38]. Education is an important social indicator, which can determine the status of a household [39]. The household’s socio-economic status, such as access to improved water, income and access to improved latrine facilities were associated with maternal education. The household’s socio-economic status can further explain the relationship between education and infant mortality [40].

The effect of maternal education on infant survival can be explained in different dimensions (economic, social demographic, environmental and biomedical) [36]. For example, maternal education can create awareness and can promote appropriate infant feeding and care [41]. Alternatively, participation in higher education enables a woman to acquire a better occupation and hence a higher income/wealth level [37].

Maternal education can also influence the attitudes of mothers towards traditional norms and beliefs including traditional infant caring practices, knowledge about illness and disease prevention practices, which have an impact on the infant and child survival [40]. An educated mother is more likely to use modern health care facilities and more aware about hygienic practices [42, 43]. Educated mothers are also give their own care during pregnancy and the care of her child through the most vulnerable stages of its life than non-educated mothers [40, 44].

Educational level can also affect infant survival by influencing the reproductive behaviour and increasing mother’s skills in health care practices related to contraception use, nutrition, hygiene, preventive care and disease treatment [45]. It may also increase chance of marrying a man in a higher occupation group and/or with a higher income, which may increase the opportunity or greater capability to provide a variety of goods, services and assets at the household level, which in turn enhances child health and survival [45]. Maternal education also increases the opportunity of a household to provide children with a sufficient amount of food, and also improves the capacity to pay health service during maternity care and childbirth [46, 47].

## Limitations

This meta-analysis may have the following limitations; as this meta-analysis included few publications, the power of this pooled effect may be small. Due to the nature of meta-analysis, that used group data, other confounding factors that can affect infant mortality were not reported. In addition to this, all studies included are observational studies that the confounding factors might present in the original studies and this may affect the overall estimate. Therefore, the findings of this study are best if considered the context of both limitations of the original studies and this meta-analysis.

## Conclusion

From this systematic review, we found that increased level of maternal education was significantly associated with infant mortality; when the level of maternal education was higher, the infant mortality was lower. From this study, investment in maternal education might be one of the important ways to reduce infant mortality in the long run; it is also one of the indicators of development of a country. Therefore, understanding the long-term effect of maternal education on infant mortality may contributes to the improvement of infant health in Ethiopia. In Ethiopia, only 4% of women completed secondary school or went beyond secondary school, obviously this calls the attention of policy makers to encourage women to go to school for longer period of time and for greater number of years. This can be done by changing the attitude of the communities, families and parents to keep women in school for longer number of years. In this regard, there should be an intersectoral collaboration between the Federal Ministry of Education and the Federal Ministry Health in Ethiopia. In addition, education programme and community information in Ethiopia should work in collaboration to promote awareness and understanding of the broad field of population issues with the purpose of developing responsible attitudes and behaviour toward that issue. Overall, policy makers should give more attention in promoting the role of women through removing institutional and cultural barriers, which hinder women from access to education in order to reduce infant mortality in Ethiopia.

## Supporting information

S1 Table Article search strategy. (DOC)

S2 Table JBI critical appraisal checklist for studies reporting analytical cross sectional studies. (DOC)

S3 Table JBI critical appraisal checklist for cohort studies. (DOC)

S4 Table JBI critical appraisal checklist for case control studies. (DOC)

S1 File Completed PRISMA 2009 checklist. (DOC)
