# Supplementary material for: The effect of maternal education on infant mortality in Ethiopia: A systematic review and meta-analysis
Source: PLoS One. 2019 Jul 29;14(7):e0220076. doi: 10.1371/journal.pone.0220076 (PMC6663004; doi:10.1371/journal.pone.0220076)
Supplement: S1 Table — (DOC) [file pone.0220076.s001.doc]

S1 Table: Search strategy for impact of maternal education on infant mortality in MEDLINE

| **#** | Searches | Result |
| --- | --- | --- |
| **1** | child mortality/ or infant mortality/ or perinatal mortality/ or neonatal mortality/ | 57162 |
| **2** | ((infant* or bab* or child* or newborn) adj3 (death* or mortalit*)).mp. | 93906 |
| **3** | 1 or 2 | 104651 |
| **4** | Educational Status/ or maternal education.mp. | 66386 |
| **5** | Education/ | 391829 |
| **6** | ((mother* or maternal) adj3 (educat* or litera* or knowlege)).mp. | 12474 |
| **7** | 4 or 5 or 6 | 455951 |
| **8** | Ethiopia | 14738 |
| **9** | 3 and 7 and 8 (limit to human and English language) | 322 |
