# Supplementary material for: The effect of maternal education on infant mortality in Ethiopia: A systematic review and meta-analysis
Source: PLoS One. 2019 Jul 29;14(7):e0220076. doi: 10.1371/journal.pone.0220076 (PMC6663004; doi:10.1371/journal.pone.0220076)
Supplement: S2 Table — (DOC) [file pone.0220076.s002.doc]

**S2 Table: JBI Critical Appraisal Checklist for Analytical Cross Sectional Studies**

Reviewer Date

Author Year Record Number

|  | Yes | No | Unclear | Not applicable |
| --- | --- | --- | --- | --- |
| 1. Were the criteria for inclusion in the sample clearly defined? | □ | □ | □ | □ |
| 1. Were the study subjects and the setting described in detail? | □ | □ | □ | □ |
| 1. Was the exposure measured in a valid and reliable way? | □ | □ | □ | □ |
| 1. Were objective, standard criteria used for measurement of the condition? | □ | □ | □ | □ |
| 1. Were confounding factors identified? | □ | □ | □ | □ |
| 1. Were strategies to deal with confounding factors stated? | □ | □ | □ | □ |
| 1. Were the outcomes measured in a valid and reliable way? | □ | □ | □ | □ |
| 1. Was appropriate statistical analysis used? | □ | □ | □ | □ |

Overall appraisal: Include □ Exclude □ Seek further info □

Comments (Including reason for exclusion)
